# Co-infection tuberculose/VIH et Maladie de kaposi sous traitement de substitution par la méthadone: à propos d’un cas

**DOI:** 10.11604/pamj.2017.28.43.11161

**Published:** 2017-09-15

**Authors:** Viviane Marie Pierre Cisse Diallo, Louise Fortes Deguenonvo, Khardiata Diallo Mbaye, Daye Ka, Ndeye Aissatou Lakhe, Ibrahima Ndiaye, Daouda Thioub, Sylvie Audrey Diop Nyafouna, Aminata Massaly, Alassane Dièye, Moustapha Diop, Cheikh Tidiane Ndour, Moussa Seydi

**Affiliations:** 1 Service des Maladies Infectieuses et Tropicales, Centre Hospitalier National Universitaire de Fann Dakar /Université Cheikh Anta Diop de Dakar, BP 5035 Dakar, Sénégal; 2 Service de Psychiatrie / Centre Hospitalier National Universitaire de Fann Dakar/Université Cheikh Anta Diop de Dakar, BP 5035 Dakar, Sénégal

**Keywords:** Tuberculose, Kaposi, VIH, méthadone, Tuberculosis, Kaposi, HIV, methadone

## Abstract

Le diagnostic tardif de l’infection à VIH peut être fatal car favorisant l’apparition d’infections opportunistes dont la prise en charge nécessite l’utilisation de plusieurs molécules pouvant provoquer des interactions médicamenteuses. Nous rapportons le cas d’une patiente de 45 ans, sous traitement de substitution à l’héroïne par la méthadone, VIH1 sous traitement antirétroviral. Cette patiente présentait un tableau pulmonaire non spécifique associant une toux quinteuse sèche avec une dyspnée d’apparition progressive évoluant dans un contexte fébrile. Par ailleurs l’examen notait un lymphœdème du membre inferieur gauche surmontée de nodules angiomateux indolores évoluant depuis trois ans associé à des plaques, des nodules angiomateux d’apparition plus récente au niveau de la face antérieure du thorax. Le GeneXpert sur les crachats avait permis d’isoler Mycobacterium tuberculosis. Le diagnostic retenu était celui d’une tuberculose pulmonaire associée à une maladie de kaposi cutané sur un terrain d’immunodépression au VIH.

## Abstract (en)

Late diagnosis of HIV infection can be fatal because it favors the appearance of opportunistic infections whose management requires the use of several molecules which can cause drug interactions. We report the case of a 45-year old female patient under heroin substitution treatment, using methadone and with HIV1 under antiretroviral treatment. This patient had nonspecific pulmonary appearance associated with dry nagging cough and progressive dyspnea evolving in a feverish context. Moreover, clinical examination showed left lower limb lymphedema with painless angiomatous nodules evolving over three years associated with plaques, angiomatous nodules occurred more recently at the level of the anterior face of the thorax. Sputum GeneXpert test allowed isolation of Mycobacterium tuberculosis. The diagnosis of pulmonary tuberculosis associated with Kaposi’s sarcoma and immunosuppression caused by HIV was retained.

## Introduction

L’infection à VIH lorsqu’elle est diagnostiquée tardivement peut entrainer la survenue de plusieurs infections opportunistes. L’association maladie de kaposi et tuberculose est peut décrite dans la littérature [1]. Le diagnostic est difficile car ces pathologies ont beaucoup de similitudes. Le traitement nécessite l’utilisation de plusieurs molécules et peut provoquer des interactions médicamenteuses. Nous rapportons les problèmes de prise en charge d’une patiente infectée par le VIH sous traitement ARV présentant l’association tuberculose/VIH et maladie de Kaposi dans un contexte de traitement de substitution par la méthadone.

## Patient et observation

**Figure 1:**
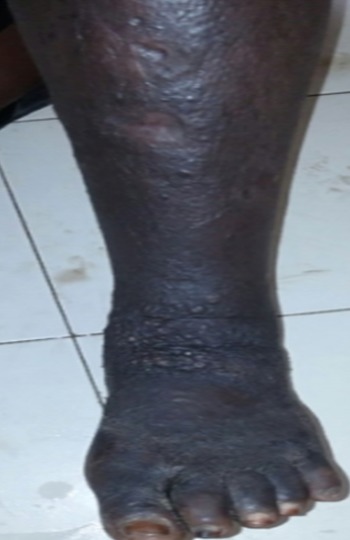
Lymphoedeme de la jambe gauche surmonté de nodules

**Figure 2:**
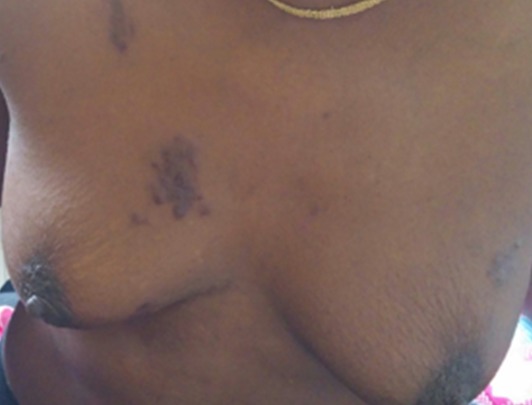
Plaques et nodules angiomateux au niveau de la face antérieure du thorax

**Figure 3:**
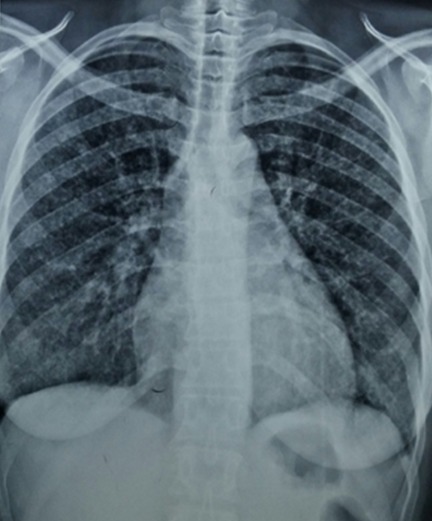
Radiographie du thorax de face montrant des micronodules disséminés de façon diffuse sur les deux champs pulmonaires associés à des opacités réticulaires

Il s’agit d’une patiente de 45 ans, VIH-1 connue depuis 13 mois, découverte deux ans après la survenue d’une maladie de kaposi cutanée sous traitement antirétroviral associant ténofovir, lamivudine et efavirenz, inobservante, admise le 22 juillet 2016 au service des Maladies Infectieuses du CHNU de Fann. Elle présentait depuis 1 mois une toux quinteuse sèche associée à une dyspnée d’apparition progressive évoluant dans un contexte de fièvre sans horaire particulière. Dans ses antécédents, on retrouvait une addiction sévère à l’héroïne depuis plus de 12 ans sous traitement de substitution par la méthadone depuis 16 mois, sans aucune notion de contage tuberculeux. À l’examen physique, elle présentait un mauvais état général, une fièvre à 39°C, une fréquence respiratoire à 25 cycles/mn et un syndrome de condensation pulmonaire bilatérale. Par ailleurs l’examen notait un lymphœdème du membre inferieur gauche remontant jusqu’à la jambe surmontée de nodules angiomateux indolores évoluant depuis trois ans (Figure 1). Au niveau de la face antérieure du thorax on notait des plaques et des nodules angiomateux d’apparition plus récente (Figure 2). La numération formule sanguine avait objectivé une leucopénie à 3000 GB/mm^3^, une anémie à 9,5 g/dl normocytaire normochrome. Concernant, l’exploration de la pneumopathie, la recherche de BAAR était négative dans les crachats alors que le GeneXpert sur les crachats avait permis d’isoler *Mycobacterium tuberculosis* sensible à la rifampicine. La radiographie du thorax montrait des micronodules disséminés de façon diffuse sur les deux champs pulmonaires associés à des opacités réticulaires avec absence de déplacement des lignes médiastinales (Figure 3). En résumé, le diagnostic retenu était celui d’une tuberculose pulmonaire associée à une maladie de kaposi cutané sur un terrain d’immunodépression au VIH. La patiente a été mise sous un traitement antituberculeux sous une quadrithérapie associant rifampicine – isoniazide - pyrazinamide et éthambutol à raison de 3 comprimés par jour. Après renforcement de l’observance le traitement antirétroviral a été poursuivi en association du traitement de substitution par la méthadone avec une augmentation progressive des doses. L’évolution a été marquée par une amélioration de son état pulmonaire avec disparition de la fièvre au bout de 5 jours, de la toux et de la dyspnée secondairement. Sur le plan cutané les lésions sont restées stables. La patiente était sortie au bout de 9 jours d’hospitalisation et continuait à être suivie en ambulatoire. Malgré une prise en charge bien menée la patiente est décédée au bout de 6 semaines.

## Discussion

L’association maladie de kaposi et tuberculose est peut décrite dans la littérature. Le tableau pulmonaire que présentait notre patiente n’était pas spécifique. Ce qui pouvait entrainer une errance diagnostique chez cette patiente qui vivait depuis 3 ans avec cette maladie de kaposi cutanée siégeant au niveau du membre inférieur gauche. Par ailleurs l’apparition récente de ces plaques et nodules angiomateux au niveau du thorax associé à ces signes respiratoires nous faisait plus penser à une dissémination de son kaposi avec localisation secondaire pulmonaire. Dans la maladie de kaposi l’atteinte pulmonaire survient dans 45% chez des patients ayant une localisation cutanée [2, 3]. Sur le plan radiologique les lésions d’une maladie de kaposi pulmonaire réalisent soit de multiples micronodules denses, homogènes souvent mal délimités, soit des opacités linéaires, bilatérales péribronchovasculaires effaçant les contours vasculaires périhilaires prédominant aux bases [4]. Les lésions observées au niveau de la radiographie du thorax de notre patiente n’écartaient pas cette hypothèse. Chez notre patiente la recherche de BAAR dans les crachats était négative et la positivité du GeneXpert dans les crachats nous a permis de poser le diagnostic de la tuberculose pulmonaire en isolant *Mycobacterium tuberculosis*. Ceci conforte les conclusions d’une étude menée dans le même service qui confirmait la sensibilité du GeneXpert par rapport à la bacilloscopie [5]. D’où l’intérêt de mieux vulgariser cet examen dans le diagnostic de la tuberculose. Le deuxième intérêt de ce dossier résidait dans la difficulté de prise en charge de ce cas. Cette patiente n’a consulté que dans le cadre de son addiction à l’héroïne, dans le contexte d’une prise en charge intégrée, son infection à VIH a été diagnostiquée deux ans après la survenue du kaposi. Ceci témoigne du retard de consultation avec comme conséquence la survenue d’une autre infection opportuniste comme ce fut le cas de notre patiente qui présentait en plus de la maladie de kaposi cutané une tuberculose pulmonaire. Avant son hospitalisation le traitement de substitution par la méthadone associée à son traitement ARV était déjà institué un peu plus d’an auparavant. En outre, cette patiente devait bénéficier des antituberculeux et d’un traitement optimal du kaposi. Plusieurs études ont montré les effets bénéfiques d´une trithérapie antirétrovirale comprenant un anti protéase dans le traitement de cette forme épidémique de la maladie de kaposi [6]. La rifampicine et les anti protéases entraînent une diminution de la concentration de la méthadone [7, 8] et peuvent avoir un impact négatif dans le suivi de ces patients sous substitution. Par ailleurs les anti protéases en plus de leur interaction avec la méthadone pouvait interagir avec la rifampicine en [9]. Chez notre patiente vu les interactions des anti protéases avec d’une part la méthadone et d’autre part la rifampicine, il a été opportun de garder le même traitement antirétroviral à savoir les deux inhibiteurs nucléosidiques et un inhibiteur non nucléosidique (ténofovir, lamivudine et efavirenz). Après l’instauration du traitement antituberculeux les doses de méthadone ont été augmentées progressivement. Le retard de consultation de cette patiente infectée par le VIH est un facteur de mauvais pronostic, car cela a engendré des difficultés dans la prise en charge globale de ces infections opportunistes. Les interactions médicamenteuses aggravent ce pronostic dans les pays à ressources limitées.

## Conclusion

Cette observation montre que la tuberculose pulmonaire demeure une infection opportuniste fréquente dans notre contexte et doit être recherchée activement. Les interactions médicamenteuses constituent un volet important à prendre en compte dans le suivi des patients car leurs effets délétères peuvent constituer un frein dans leur prise en charge.

## Conflits d’intérêts

Les auteurs ne déclarent aucun conflit d'intérêts.
